# Current status and etiology of valvular heart disease in China: a population-based survey

**DOI:** 10.1186/s12872-021-02154-8

**Published:** 2021-07-13

**Authors:** Ying Yang, Zengwu Wang, Zuo Chen, Xin Wang, Linfeng Zhang, Suning Li, Congyi Zheng, Yuting Kang, Linlin Jiang, Zhenhui Zhu, Runlin Gao

**Affiliations:** 1 Division of Prevention and Community Health, National Center for Cardiovascular Disease, National Clinical Research Center of Cardiovascular Disease, State Key Laboratory of Cardiovascular Disease, Fuwai Hospital, Peking Union Medical College & Chinese Academy of Medical Sciences, No. 15 (Lin), Fengcunxili, Mentougou District, Beijing, 102308 China; 2 Department of Echocardiography, Fuwai Hospital, Peking Union Medical College & Chinese Academy of Medical Sciences, Beijing, 100037 China; 3 Department of Cardiology, Fuwai Hospital, Peking Union Medical College & Chinese Academy of Medical Sciences, No. 167, Beilishilu, Xicheng District, Beijing, 100037 China

**Keywords:** China, Degenerative, Prevalence, Rheumatic, Valvular heart disease

## Abstract

**Background:**

The epidemiology of valvular heart disease (VHD) has changed markedly over the last 50 years worldwide, and the prevalence and features of VHD in China are unknown. The objective of this study was to investigate the current status and etiology of VHD in China.

**Methods:**

We used a cross-sectional national survey with stratified multistage random sampling from the general Chinese population to estimate the VHD burden. Data on demographic characteristics, medical history, physical examination, blood tests, and potential etiology were collected. Echocardiography was used to detect VHD.

**Results:**

The national survey enrolled 34,994 people aged 35 years or older across China. Overall, 31,499 people were included in the final analysis, and 1309 participants were diagnosed with VHD. The weighted prevalence was 3.8%, with an estimated 25 million patients in China. The prevalence of VHD increased with age and was higher in participants with hypertension or chronic kidney disease than in their counterparts. Among participants with VHD, 55.1% were rheumatic and 21.3% were degenerative. The proportion of rheumatic decreased with age, and the proportion of degenerative rose with age. However, the prevalence of rheumatic disease was still higher in the elderly population than in the younger population. Logistic regression revealed that age and hypertension were correlated with VHD.

**Conclusions:**

In China, rheumatic heart disease was still the major cause of the VHD, with a significant increase in degenerative heart disease. Age and hypertension are important and easily identifiable markers of VHD.

**Supplementary Information:**

The online version contains supplementary material available at 10.1186/s12872-021-02154-8.

## Introduction

Valvular heart disease (VHD) is mainly caused by rheumatic heart disease (RHD) or occurs as a consequence of aging (degenerative) worldwide. The epidemiology of VHD has changed markedly over the last 50 years worldwide. RHD remains a major problem in developing countries [1], but most VHDs in industrialized countries are degenerative [2, 3]. China was once estimated to have a large number of patients with RHD. After great improvements in the Chinese economy and people’s living conditions since the policy of reformation and opening in 1978, infectious diseases, as well as RHD, have declined significantly, while chronic diseases have increased at the same time [4]. It is speculated that valvular degeneration is increasing with the accelerated demographic aging tendency in China [5]. The current status of degenerative heart disease (DHD) is uncertain.

There are few data on the prevalence of VHD, especially in developing countries, in contrast to the many studies on percutaneous interventional techniques in this field. The main difficulty in obtaining such data is the requirement of high-quality echocardiographic examinations in a large, representative sample. In a pooled population-based study in 2006, Nkomo et al*.* reported that the prevalence of VHD was 2.5% in American adults [6]. In the UK, a recent population-based study reported a 51% VHD prevalence in the older population [7]. Previous studies conducted in hospitals or a single province in China reported limited information on the etiology and severity of VHD [8–10]. The aim of this study, therefore, is to assess the current status and etiology of VHD from the echocardiography data of a large nationwide population sample.

## Methods

### Study population

This was a prospective cross-sectional study conducted between October 2012 and December 2015. A multistage random sampling method was used to obtain a nationwide sample representing the general population over 15 years old across all 31 provinces in the China hypertension survey [11, 12]. Permanent residents randomly chosen from 262 urban cities and rural counties were enrolled. To further study VHD prevalence, all 262 selected urban and rural areas were stratified into eastern, middle, and western regions to represent both geographical location and economic level. Using the simple random sampling method, 16 cities and 17 counties were selected, including 7 cities and 7 counties in the eastern region, 6 cities and 6 counties in the middle region, and 3 cities and 4 counties in the western region. Then, at least three communities or villages were randomly selected from each city or county. To meet the designed sample size of 35,000 participants aged ≥ 35 years and taking nonresponses into account, 56,000 subjects were randomly selected from the eligible sites. Finally, 34,994 participants completed the survey, for an overall response rate was 62.5%. After excluding subjects without demographic information (n = 627), laboratory tests (n = 1 257), and inadequate echocardiographic images (n = 1611), 31,499 subjects were included in the final analysis.

### Data collection

A standardized questionnaire was specifically developed for this study to collect demographic characteristics, lifestyle and history of disease by experienced medical staff. The history of cardiac diseases, such as myocardial infarction, coronary artery bypass grafting surgery, percutaneous coronary intervention, congestive heart failure and so on, was documented in detail. For the purpose of identifying comorbidities, all instances of hypertension, stroke, hyperlipidemia, diabetes mellitus, and chronic kidney disease (CKD) were also recorded in the questionnaire. Subsequently, physical examination, electrocardiogram, and echocardiography were performed on each participant at the local medical centers (town/county hospitals). Blood samples were collected to test for blood lipids, glucose, and creatinine in a designated lab.

Hypertension was defined as systolic blood pressure (BP) ≥ 140 mmHg and/or diastolic BP ≥ 90 mmHg and/or the use of antihypertensive medication within the last two weeks. Diabetes was defined as fasting plasma glucose level ≥ 7.0 mmol/L and/or taking hypoglycemic agents or insulin. Dyslipidemia was defined as total cholesterol ≥ 6.22 mmol/L, low-density lipoprotein cholesterol (LDL-C) ≥ 4.14 mmol/L, high-density lipoprotein cholesterol (HDL-C) < 1.04 mmol/L, triglycerides ≥ 2.26 mmol/L, a combination thereof, or taking lipid-lowering drugs. CKD was defined as decreased kidney function, expressed as an estimated glomerular filtration rate < 60 ml/min/1.73 m^2^ by the MDRD equation or a ratio of albumin and creatinine in a urine sample ≥ 30 mg/mmol [13, 14]. AF was verified by the current ECG report or verified AF history. Participants were diagnosed with systolic heart failure(HF) based on symptoms and a transthoracic echocardiogram showing left ventricular ejection fraction (LVEF) < 50% or medical records issued by a local county or higher-level hospital.

### Echocardiography procedures

Transthoracic echocardiography was performed in the participant’s local medical center by experienced sonographers who were trained uniformly to quantify cardiac chambers according to the requirements of this study. Two experts from Fuwai Hospital supervised the field investigation in each local medical center. 2D images were acquired and measured in the parasternal (standard long- and short-axis images) and apical views (2-, 4-chamber, and apical long-axis images). The assessment of valvular stenosis and regurgitation by Doppler was carried out as recommended by the American Society of Echocardiography [15, 16]. Aortic stenosis was classified as none, mild, moderate and severe according to the highest aortic jet velocities: < 2.6 m/s, 2.6–2.9 m/s, 3.0–4.0 m/s and > 4.0 m/s, respectively. A subject with aortic maximal jet velocity ≥ 2.6 m/s was diagnosed with aortic stenosis (AS) and was enrolled in the current study. Subjects with a mitral valve area ≤ 2.0 cm^2^ were defined as having mitral stenosis (MS). Comprehensive measurements, including quantitative and semiquantitative methods, are commonly used for evaluating valvular regurgitation. All images and data were stored in a database and transferred via the internet to the coordinating centers to be evaluated by experts on the image quality and measurement accuracy. Discrepancies were resolved to achieve a mutual consensus at the same time.

VHD was confined to findings of mild or more severe stenosis and moderate or more severe regurgitation, consistent with the pioneer study in Europe [17]. A valve involved with stenosis and/or regurgitation was considered valvular dysfunction. Any participant with multiple valvular dysfunctions was counted only once in the VHD definition. RHD was diagnosed definitely by the 2012 World Heart Federation criteria, according to the pathological valvular dysfunctions in the left heart and specific morphological features (for example, mitral valve leaflet thickening) [18]. DHD required the presence of mitral and/or aortic leaflet sclerosis and calcification in the absence of rheumatic features (such as commissural fusion) and valvular congenital abnormalities among those above 60 years old, regardless of the functional status. The DHD also covered mitral valve prolapse. Valve replacement was confirmed by medical history and detection of the prosthetic valve by echocardiography. Congenital heart diseases (CHDs) included atrial septal defects, ventricular septal defects, pulmonary stenosis, bicuspid and quadricuspid aortic valves, double-orifice mitral valves, and persistent left superior vena cava. Ischemia was verified by medical history with typical electrocardiographic changes (Q wave) or echocardiographic detection of myocardial ischemia, which is based on visualizing a regional decrease in systolic endocardial motion and myocardial thickening. The causes of secondary VHD included ischemia, nonvalvular atrial fibrillation (AF), HF, dilated cardiomyopathy, hypertrophic cardiomyopathy, and CHDs except for congenital valvular anomalies. Some VHDs had unclear etiologies based on available information and were classified as “other”.

### Statistics

All data in the analysis were weighted based on the 2010 China population census data [19]. The demographic and clinical characteristics were collected from participants with and without VHD. Descriptive statistics for the study are presented as means with 95% confidence intervals (CIs) for continuous variables and percentages with 95% CIs for categorical variables. Student’s *t*-test and the c*hi*-squared test or Fisher’s exact test were used to compare groups with and without VHD. The prevalence of major valvular dysfunctions was stratified by age, sex and comorbidities. A bar graph was used to show the distribution of etiologies in different age strata. A logistic regression model was used to assess associations between VHD and demographic characteristics. The variables adjusted and results expressed as odds ratios (ORs) with 95% CIs are listed in the forest plot. A two-tailed *P* value < 0.05 was considered significant. Statistical analyses were conducted with SAS version 9.4 (SAS Institute INC, Cary, NC, USA).

## Results

Of all 31,499 participants, the mean age was 51.8 years, and 49.3% were female (Table 1). The participants with VHD were older than those without VHD, and 48.2% were male. All diseases and comorbidities were different (*P* < 0.001) between the VHD and non-VHD groups except for diabetes.

**Table 1:** Characters of the study population with and without valvular heart disease

	Total (n = 31,499)	VHD (n = 1309)	No VHD (n = 30,190)	*P* value
Gender				0.308
Male (%)	50.7 (49.0–52.5)	48.2 (42.9–53.5)	50.8 (49.0–52.7)	
Female (%)	49.3 (47.5–51.0)	51.8 (46.5–57.1)	49.2 (47.3–51.0)	
Age (mean, years)	51.8 (51.0–52.5)	61.6 (59.9–63.2)	51.4 (50.7–52.1)	< 0.001
BMI (mean, Kg/m^2^)	24.7 (24.3–25.0)	24.2 (23.5–24.9)	24.7 (24.3–25.1)	0.058
Region				0.134
East (%)	44.1 (20.1–68.2)	49.2 (15.4–82.9)	43.9 (20.1–67.8)	
Central (%)	34.5 (11.0–58.0)	21.7 (7.8–47.8)	35.0 (11.3–58.7)	
West (%)	21.4 (8.0–45.9)	29.1 (0.0–61.7)	21.0 (7.9–45.2)	
Residence				0.407
Rural (%)	37.5 (22.3–52.7)	44.3 (15.1–73.7)	37.2 (22.4–52.0)	
Urban (%)	62.5 (47.3–77.7)	55.7 (26.4–84.9)	62.8 (48.0–77.6)	
Heart diseases history				
Ischemia (%)	0.5 (0.2–1.0)	1.9 (0.8–4.8)	0.4 (0.2–0.9)	< 0.001
AF (%)	0.7 (0.5–1.1)	4.5 (2.9–6.9)	0.6 (0.4–0.9)	< 0.001
Systolic HF (%)	0.8 (0.6–1.1)	3.9 (2.6–5.9)	0.7 (0.5–1.0)	< 0.001
DCM (%)	0.3 (0.2–0.5)	3.5 (1.8–6.7)	0.2 (0.1–0.3)	< 0.001
HCM (%)	0.2 (0.1–0.4)	0.8 (0.3–2.0)	0.2 (0.1–0.3)	< 0.001
CHD (%)	0.2 (0.1–0.4)	1.4 (0.6–3.3)	0.1 (0.0–0.3)	< 0.001
Comorbidities				
Hypertension (%)	35.1 (32.8–37.4)	51.9 (48.4–55.4)	34.4 (32.2–36.7)	< 0.001
Stroke (%)	1.6 (1.0–2.4)	3.3 (1.8–6.1)	1.5 (1.0–2.3)	0.004
Dyslipidemia (%)	32.4 (28.4–36.4)	28.9 (23.6–34.1)	32.5 (28.5–36.5)	0.015
Diabetes (%)	8.5 (7.5–9.7)	9.0 (7.5–10.8)	8.5 (7.4–9.8)	0.581
CKD (%)	4.6 (3.7–5.7)	11.0 (8.4–14.2)	4.3 (3.5–5.3)	< 0.001
Echocardiography measurements				
LAap (mm)	31.2 (30.4–32.1)	34.3 (32.9–35.7)	31.1 (30.3–32.0)	< 0.001
LVEDD (mm)	46.2 (45.5–47.0)	48.2 (47.2–49.2)	46.2 (45.4–46.9)	< 0.001
IVSd (mm)	9.6 (9.3–9.8)	10.0 (9.4–10.6)	9.5 (9.2–9.8)	0.077
LVESD (mm)	30.0 (29.3–30.8)	32.2 (31.1–33.3)	30.0 (29.2–30.7)	< 0.001
LVEF (%)	64.4 (62.8–65.9)	62.7 (60.7–64.7)	64.4 (62.9–66.0)	< 0.001

Data were represented as means or percentage (95% CI)

All values were weighted to represent the total population of Chinese aged 35 years or older based on the Chinese census 2010

VHD, valvular heart disease; BMI, body mass index; AF, atrial fibrillation; HF, heart failure; DCM, Dilated Cardiomyopathy; HCM, Hypertrophic Cardiomyopathy; CHD, congenital heart disease; CKD, chronic kidney disease. LAap, the anteroposterior diameter of the left atrium; LVEDD, the diameter of the left ventricle at end-diastole; IVSd, the thickness of the interventricular septum; LVESD, the left ventricle end-systolic diameter; LVEF, the left ventricle ejection fraction

*P* value for t-test (continuous) and x^2^ or Fisher’s exact test (categorical) to assess the difference between population with VHD and those without it

The weighted prevalence of VHD was 3.8% (95% CI 2.6–5.6, Table 2) out of an estimated 25,621,503 patients in China according to the 2010 census data. The prevalence of VHD, which increased with age (*P* < 0.001), was not different between males and females (*P* = 0.308) and was higher in participants with hypertension (5.6% vs 2.8%, *P* < 0.001) and CKD (9.2% vs 3.5%, *P* < 0.001) than in their counterparts. Overall, the most common VHD was aortic regurgitation (AR, 1.2%), followed by mitral regurgitation (MR, 1.1%), tricuspid regurgitation (TR, 0.8%), and MS (0.8%).

**Table 2:** Prevalence of valvular heart diseases (VHD) in the left heart according to characteristics

Characters	Number	VHD	MS	MR	AS	AR
Total	31,499	3.8 (2.6–5.6)	0.8 (0.5–1.3)	1.1 (0.7–1.7)	0.7 (0.4–1.1)	1.2 (0.7–2.1)
Gender						
Male	14,470	3.6 (2.5–5.2)	0.8 (0.4–1.6)	1.0 (0.7–1.4)	0.8 (0.4–1.4)	1.3 (0.8–2.2)
Female	17,029	4.0 (2.6–6.1)	0.8 (0.5–1.3)	1.2 (0.7–2.1)	0.6 (0.3–1.0)	1.2 (0.7–2.0)
*P*		0.308	0.967	0.096	0.194	0.459
Age (years)						
35–44	6849	1.9 (1.1–3.2)	0.7 (0.3–1.5)	0.4 (0.2–0.7)	0.4 (0.2–0.7)	0.3 (0.1–0.8)
45–54	7406	2.1 (1.4–3.0)	0.5 (0.3–1.0)	0.7 (0.5–1.0)	0.3 (0.1–0.8)	0.4 (0.3–0.8)
55–64	6765	4.5 (3.0–6.8)	0.7 (0.4–1.2)	1.5 (0.9–2.4)	0.5 (0.3–1.0)	1.4 (0.8–2.6)
65–74	6406	7.6 (5.1–11.2)	1.4 (0.7–2.5)	2.1 (1.3–3.3)	1.5 (0.8–2.8)	3.1 (1.7–5.7)
75-	4073	15.9 (10.3–23.7)	2.1 (1.1–3.8)	5.2 (3.2–8.3)	3.4 (1.7–6.7)	7.1 (4.0–12.3)
*P*		< 0.001	0.002	< 0.001	< 0.001	< 0.001
BMI (Kg/m^2^)						
< 18.5	1319	6.2 (3.7–10.4)	1.6 (0.6–4.7)	1.7 (0.6–4.4)	0.7 (0.2–1.8)	3.1 (1.6–6.1)
18.5–23.9	13,036	4.0 (2.6–6.0)	0.7 (0.4–1.4)	1.2 (0.8–1.8)	0.7 (0.3–1.3)	1.2 (0.6–2.1)
24–27.9	12,042	3.7 (2.4–5.5)	0.8 (0.5–1.3)	1.1 (0.6–1.9)	0.7 (0.4–1.1)	1.2 (0.7–1.9)
28-	5102	3.1 (1.9–5.0)	0.9 (0.5–1.5)	0.9 (0.5–1.5)	0.6 (0.3–1.4)	1.1 (0.6–2.0)
*P*		0.042	0.286	0.583	0.996	0.001
Region						
East	14,009	4.2 (2.5–7.1)	0.9 (0.5–1.8)	1.0 (0.6–1.5)	0.8 (0.4–1.6)	1.8 (1.0–3.3)
Central	11,895	2.4 (1.0–5.9)	0.4 (0.1–0.9)	1.1 (0.4–2.9)	0.2 (0.1–0.6)	0.6 (0.2–2.1)
West	5595	5.2 (2.8–9.5)	1.2 (0.4–4.3)	1.4 (0.5–3.9)	1.1 (0.3–3.5)	1.1 (0.6–2.0)
*P*		0.134	0.070	0.800	0.022	0.022
Residence						
Rural	15,009	4.5 (2.5–7.9)	0.9 (0.4–1.9)	1.3 (0.7–2.4)	0.8 (0.4–1.6)	1.4 (0.7–2.9)
Urban	16,490	3.4 (2.0–5.6)	0.7 (0.4–1.5)	1.0 (0.6–1.7)	0.6 (0.3–1.3)	1.1 (0.5–2.3)
*P*		0.407	0.636	0.551	0.603	0.629
Hypertension						
No	18,163	2.8 (1.9–4.2)	0.6 (0.4–1.1)	0.8 (0.5–1.3)	0.4 (0.2–0.6)	0.7 (0.4–1.2)
Yes	13,336	5.6 (3.8–8.3)	1.1 (0.7–1.8)	1.6 (1.0–2.4)	1.2 (0.6–2.3)	2.2 (1.3–3.6)
*P*		< 0.001	< 0.001	< 0.001	< 0.001	< 0.001
Dyslipidemia						
No	21,830	4.0 (2.7–5.9)	0.8 (0.5–1.4)	1.1 (0.8–1.7)	0.7 (0.4–1.1)	1.3 (0.8–2.2)
Yes	9669	3.4 (2.2–5.1)	0.7 (0.4–1.3)	1.0 (0.6–1.8)	0.6 (0.3–1.2)	1.0 (0.6–1.8)
*P*		0.015	0.700	0.549	0.791	0.025
Diabetes						
No	28,214	3.8 (2.6–5.6)	0.8 (0.5–1.3)	1.1 (0.7–1.6)	0.6 (0.4–1.0)	1.2 (0.7–2.1)
Yes	3285	4.0 (2.5–6.3)	1.0 (0.6–1.9)	1.5 (0.8–2.9)	1.2 (0.5–2.6)	1.3 (0.7–2.2)
*P*		0.581	0.310	0.131	0.018	0.889
Stroke						
No	30,656	3.7 (2.5–5.5)	0.8 (0.5–1.3)	1.1 (0.7–1.7)	0.6 (0.4–1.1)	1.2 (0.7–2.0)
Yes	843	7.9 (4.0–15.1)	1.3 (0.4–3.8)	1.9 (0.8–4.7)	1.8 (0.7–4.6)	4.1 (2.2–7.8)
*P*		0.004	0.295	0.185	0.003	< 0.001
CKD						
No	29,453	3.5 (2.4–5.2)	0.7 (0.4–1.2)	1.0 (0.7–1.5)	0.6 (0.4–1.0)	1.1 (0.7–1.9)
Yes	2046	9.2 (6.0–13.9)	2.3 (1.3–4.0)	3.0 (1.7–5.1)	1.6 (0.7–3.5)	3.2 (1.5–6.5)
*P*		< 0.001	< 0.001	< 0.001	0.001	< 0.001

Data were represented as a percentage (95% CI)

All values were weighted to represent the total population of Chinese aged 35 years or older based on the Chinese census 2010

VHD, valvular heart disease; MS, mitral stenosis; MR, mitral regurgitation; AS, aortic stenosis; AR, aortic regurgitation; BMI, body mass index; CKD, chronic kidney disease

*P* value for x^2^ or Fisher’s exact test to assess the difference of prevalence among subgroups according to the characteristics

**Fig. 1:**
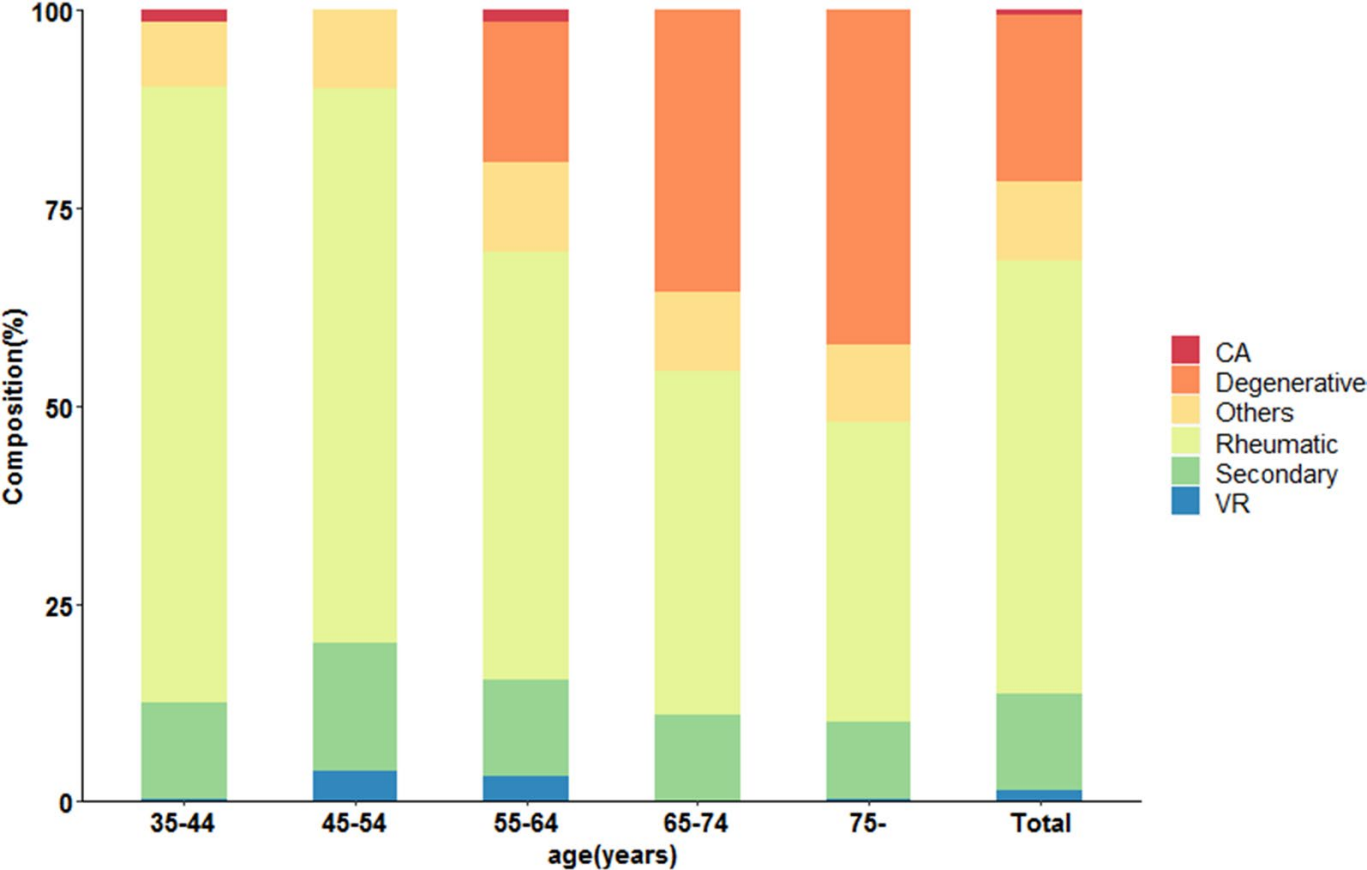
The proportions of etiologies of valvular heart disease according to age (The related dataset were seen in Additional file 1: Table S2). CA = congenital anomaly; VR = valve replacement

**Fig. 2:**
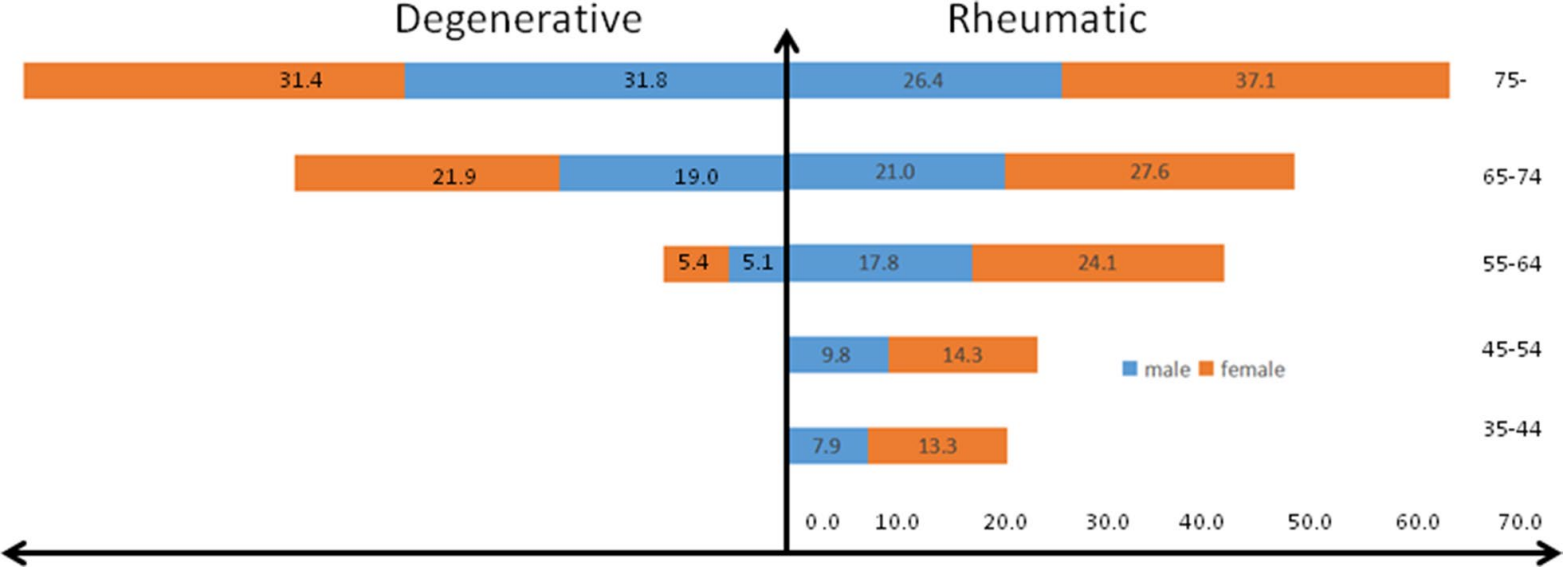
The prevalences of VHD due to the two common etiologies in different gender and age. The prevalence were reported as number × 10^–3^ without weight

**Fig. 3:**
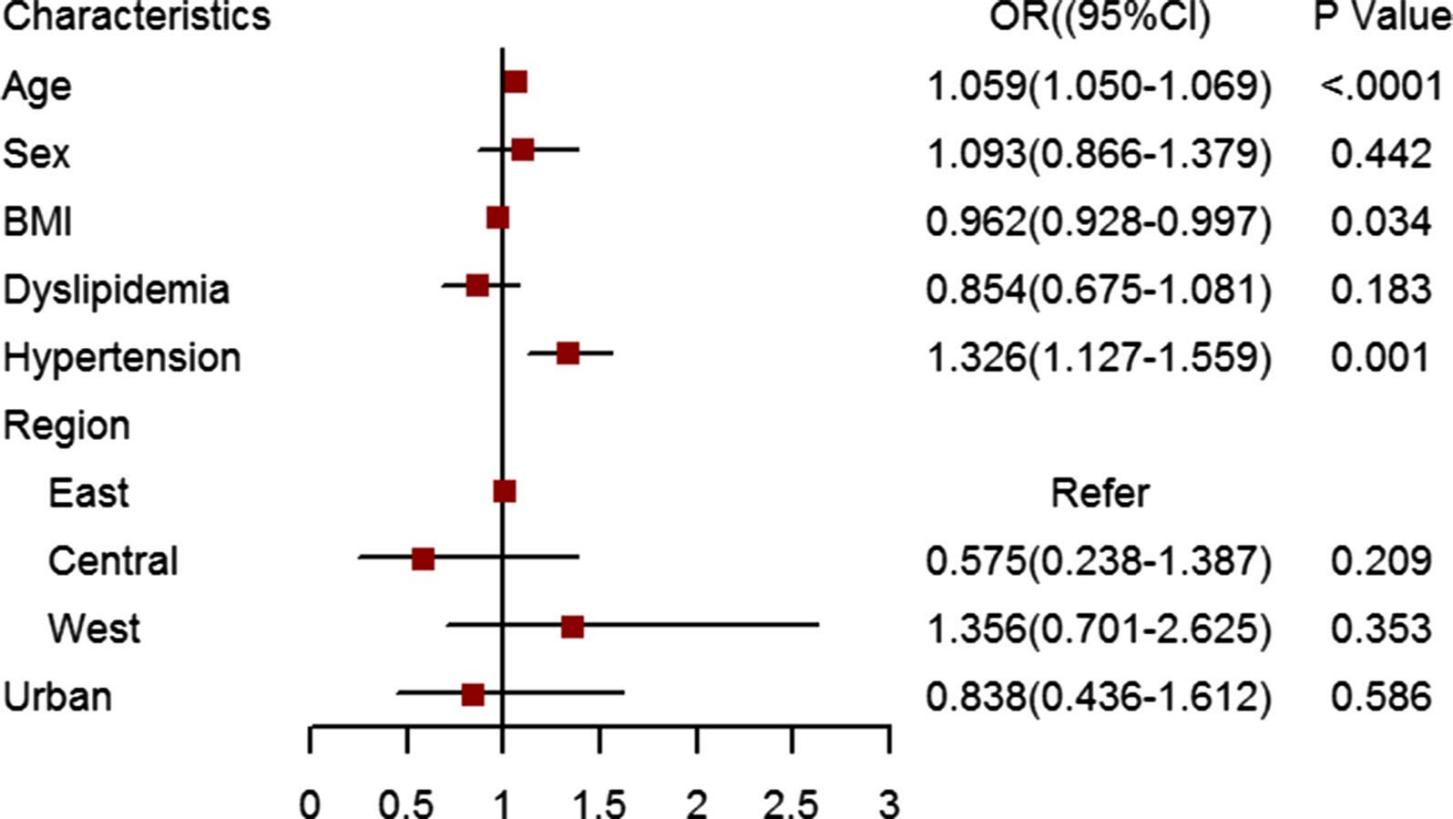
Factors related to valvular heart disease calculated by logistic regression (BMI = body mass index)

The etiologies of VHD stratified by age are shown in Fig. 1 (data listed in Additional file 1: Table S2). The proportion of RHD was 55.1%. It dropped from 77.8% in those aged 35–44 to 38.0% in those aged ≥ 75 years. The proportion of DHD was 21.3%, rising from 18.2% in the 55–64 age group to 42.5% in those aged 75 years above. Secondary VHD accounted for 12.1%, while the valve replacement etiology made up 0.9%. The crude prevalence of VHD due to the two common etiologies in different sexes and ages is shown in Fig. 2. The inverted pyramid showed that the prevalence of RHD in the elderly (2.64% in men and 3.71% in women) was apparently higher than that in those aged 35–54 (0.79% in men and 1.33% in women). Multiple logistic regression was employed to explore the associations between demographic characteristics and VHD (Fig. 3). VHD was positively associated with age and hypertension and negatively correlated with BMI.

Single aortic dysfunction (n = 496), including stenosis and/or regurgitation, was the most frequent type of valvular dysfunction (Additional file 1: Fig. S1), followed by single mitral dysfunction (n = 367). The most common double dysfunction was aortic valve disease combined with mitral valve (n = 132), and the most common triple dysfunction involved the aortic, mitral, and tricuspid valves (n = 35).

## Discussion

The current study aimed to describe the prevalence of VHD in a large sample from the general population of China. The results revealed that VHD is common and increases with age. Furthermore, RHD remains the leading cause of valvular dysfunction, and DHD is becoming an increasing problem in China. These findings suggest a tremendous burden of VHD due to a large population that is rapidly aging, and they call for echocardiographic screening.

The VHD prevalence of 2.5% in the US was lower than that in the current study [6]. The discrepancy could be attributed to different definitions (including only moderate or severe valvular stenosis and not mild stenosis) and the inclusion of more young participants. Another study was conducted among 2500 individuals aged ≥ 65 years, in which the prevalence (51%) was rather high [7]. Before the current study, the prevalence of VHD in China has not yet been determined based on available information from hospitals [8, 20]. Our results revealed that the prevalence was sensitive to age, the same as in the Western world.

The marked decrease in the prevalence of rheumatic disease in Western countries has been largely counterbalanced by the increased prevalence of DHD with the aging of the population [21]. China’s situation is slightly different. A study from a single cardiovascular center in southern China showed that the prevalence of RHD decreased significantly, but the prevalence of congenital VHD, ischemic VHD, and DHD increased markedly; however, RHD remained the leading etiology in southern China [8]. In 2019, a report showed that rheumatic origin was still the predominant etiology of left-sided valve disease in Chinese patients hospitalized for VHD [22]. The current results are in line with previous studies. Unlike in the West, rheumatic origin is the most frequent etiology, followed by degenerative causes, in China, Uganda and Tunisia [23, 24]. In our study, the burden of RHD was mainly derived from the older population, especially those aged 75 years and older. With the rapid development of the economy and improvement of residents’ living conditions in the past 40 years, the incidence of RHD has been significantly reduced in China [4, 25, 26]. Young people are unlikely to be affected, and patients with RHD are aging. In brief, RHD and DHD are both major health threats to Chinese old people and deserve more attention.

In 2007, the Euro Heart Survey, which included 5001 patients from 92 medical centers in 25 European countries, reported that AS and MR accounted for 43.1% and 33.6%, respectively, of single-valve diseases and were mostly caused by degenerative diseases [27]. The subsequent survey in 2017 further highlighted the findings of the previous results on VHD [21]. The same increasing trend for AS and MR was also demonstrated in Sweden, the UK and the US [6, 7, 28]. However, most studies in China, including ours, have shown that regurgitation is the main burden rather than aortic stenosis [20, 22, 29]. Compared with stenosis, the symptom of chronic regurgitation is likely to be unspecific, while compared to MR, the symptom of AR tends to be hidden because of the strong ability of the left ventricle to compensate for the overload of end-diastolic volume. Therefore, patients with underlying single AR might not check with their doctors in a timely manner. This might explain the relatively low rate of AR in symptomatic patients admitted to the hospital. The results of the current study suggest that the prevalence of AR among hospitalized patients might be underestimated.. The multiple involvement of degenerative changes (aging, hypertension and atherosclerosis) and RHD might be another reason for the high prevalence of AR. Hypertension accelerates degenerative changes in the aortic valve and annulus and might be the result of AR [30]. In addition to AR, age and hypertension are also observed to exert an influence on the prevalence of MR. The mechanism is longstanding mechanical stress and increased afterload, respectively, as documented by the Framingham study [31]. Regurgitation accounts for the majority of VHD cases, corresponding to the aging of the population and the high incidence of hypertension in China. The study above [31] reported that the prevalence of MR and TR decreased as an increasing BMI. The authors speculated poor ultrasonic penetration and inadequate detection of Doppler signals. The inverse relation between BMI and VHD was shown in the current results. The similar effects called obesity paradox had been shown in many chronic diseases [32]. Besides the technical factors, there were several potential mechanisms through which the paradox could arise [33]. However, there were also evidence that intentional weight loss remains protective in the patients with higher BMI.

The prevalence of any valve disease increases with age [34]. In 2010, there were 258 million people aged 55 years old and above in China, and the population aging is progressively increasing. In the future, there will be a much larger elderly population presenting with VHD. VHD is frequently diagnosed in old patients with clinical complications, such as HF, and common comorbidities, such as diabetes and coronary artery disease, which also increase the risks of treatment.

VHD is a progressive disease that may be silent for decades. It has been reported that early identification and treatment could avoid and delay heart failure from long-term overvolume and overpressure. Auscultation is a widely available method to detect VHD in large populations, but its sensitivity is limited [35]. Therefore, echo screening for early identification and prevention, as recommended by the WHO [36] in the 1980s, might also be helpful given the aging tendency.

The study has several limitations. First, this is a cross-sectional study, which means that some patients with acute or severe VHD, such as infective endocarditis, chordae rupture, and latrogenic injuries, were not included. Second, although the investigators have done their best to confirm the causes of VHD, it is hard to attribute all VHDs to clear etiologies. Third, the prevalence of VHD is associated with economic development. The income of the participants is hard to obtain in most epidemiological studies. Instead, the different regions are used to represent the economic levels in China. Normally, the economic level is lowest in the Western region, middle in the Central region, and highest in the East region. However, the current results did not reveal the difference in the prevalence of VHD among regions.

## Conclusion

To the best of our knowledge, this is the first population-based study aimed at estimating the prevalence of VHD by echocardiography in China. AR followed by MR is the most frequent valvular dysfunction in China. Despite a significant decrease of rheumatic fever in the past years, it has shown that RHD remains the leading etiology in China, but there has been a significant increase in DHD. Age and hypertension are important and easily identifiable markers for screening and preventing VHD.

## Supplementary Information

**Additional file 1** More results of the analysis.

**Additional file 2** The full list of work staff for the survey.

## Data Availability

The dataset analyzed during the current study is available from the corresponding author on reasonable request.

## Abbreviations

AF: Atrial fibrillation
AR: Aortic regurgitation
AS: Aortic stenosis
BP: Blood pressure
CHD: Congenital heart diseases
CI: Confidence intervals
CKD: Chronic kidney disease
DHD: Degenerative heart disease
HDL-C: High-density lipoprotein cholesterol
HF: Heart failure
LDL-C: Low-density lipoprotein cholesterol
LVEF: Left ventricular ejection fraction
MR: Mitral regurgitation
MS: Mitral stenosis
RHD: Rheumatic heart disease
TR: Tricuspid regurgitation
VHD: Valvular heart disease

## References

[1] Watkins DA, Johnson CO, Colquhoun SM, Karthikeyan G, Beaton A, Bukhman G, Forouzanfar MH, Longenecker CT, Mayosi BM, Mensah GA, Nascimento BR, Ribeiro ALP, Sable CA, Steer AC, Naghavi M, Mokdad AH, Murray CJL, Vos T, Carapetis JR, Roth GA (2017). Global, regional, and national burden of rheumatic heart disease, 1990–2015. New Engl J Med.

[2] Iung B, Vahanian A (2014). Epidemiology of acquired valvular heart disease. Can J Cardiol.

[3] Ray S (2010). Changing epidemiology and natural history of valvular heart disease. Clin Med (Lond).

[4] Cheng TO (2009). How much of the recent decline in rheumatic heart disease in China can be explained by changes in cardiovascular risk factors?. Int J Cardiol.

[5] Kodali SK, Velagapudi P, Hahn RT, Abbott D, Leon MB (2018). Valvular heart disease in patients ≥80 years of age. J Am Coll Cardiol.

[6] Nkomo VT, Gardin JM, Skelton TN, Gottdiener JS, Scott CG, Enriquez-Sarano M (2006). Burden of valvular heart diseases: a population-based study. Lancet.

[7] D'Arcy JL, Coffey S, Loudon MA, Kennedy A, Pearson-Stuttard J, Birks J, Frangou E, Farmer AJ, Mant D, Wilson J, Myerson SG, Prendergast BD (2016). Large-scale community echocardiographic screening reveals a major burden of undiagnosed valvular heart disease in older people: the OxVALVE Population Cohort Study. Eur Heart J.

[8] Liu FZ, Xue YM, Liao HT, Zhan XZ, Guo HM, Huang HL, Fang XH, Wei W, Rao F, Deng H, Liu Y, Lin WD, Wu SL (2014). Five-year epidemiological survey of valvular heart disease: changes in morbidity, etiological spectrum and management in a cardiovascular center of Southern China. J Thorac Dis.

[9] Shu C, Chen S, Qin T, Fu Z, Sun T, Xie M, Zhang L, Dong N, Yin P (2016). Prevalence and correlates of valvular heart diseases in the elderly population in Hubei. China Sci Rep.

[10] Wang YT, Tao J, Maimaiti A, Adi D, Yang YN, Li XM, Ma X, Liu F, Chen BD, Ma YT (2017). Prevalence of valvular heart diseases and associated risk factors in Han, Uygur and Kazak population in Xinjiang, China. PLoS ONE.

[11] Wang Z, Chen Z, Zhang L, Wang X, Hao G, Zhang Z, Shao L, Tian Y, Dong Y, Zheng C, Wang J, Zhu M, Weintraub WS, Gao R (2018). Status of hypertension in China: results from the China Hypertension Survey, 2012–2015. Circulation.

[12] Wang Z, Chen Z, Wang X, Zhang L, Li S, Tian Y, Shao L, Hu H, Gao R (2018). The disease burden of atrial fibrillation in China from a national cross-sectional survey. Am J Cardiol.

[13] Foundation NK (2002). K/DOQI clinical practice guidelines for chronic kidney disease: evaluation, classification, and stratification. Am J Kidney Dis.

[14] Lopea-Vargas PA, Tong A, Sureshkumar P, Johnson DW, Craig JC (2013). Prevention, detection and management of early chronic kidney disease: a systematic review of clinical practice guidelines. Nephrology.

[15] Baumgartner H, Hung J, Bermejo J, Chambers JB, Edvardsen T, Goldstein S, Lancellotti P, LeFevre M, Miller FJ, Otto CM (2017). Recommendations on the echocardiographic assessment of aortic valve stenosis: a focused update from the European Association of Cardiovascular Imaging and the American Society of Echocardiography. J Am Soc Echocardiogr.

[16] Zoghbi WA, Adams D, Bonow RO, Enriquez-Sarano M, Foster E, Grayburn PA, Hahn RT, Han Y, Hung J, Lang RM, Little SH, Shah DJ, Shernan S, Thavendiranathan P, Thomas JD, Weissman NJ (2017). Recommendations for noninvasive evaluation of native valvular regurgitation: a report from the American society of echocardiography developed in collaboration with the society for cardiovascular magnetic resonance. J Am Soc Echocardiogr.

[17] Iung B, Vahanian A (2011). Epidemiology of valvular heart disease in the adult. Nat Rev Cardiol.

[18] Reményi B, Wilson N, Steer A, Ferreira B, Kado J, Kumar K, Lawrenson J, Maguire G, Marijon E, Mirabel M, Mocumbi AO, Mota C, Paar J, Saxena A, Scheel J, Stirling J, Viali S, Balekundri VI, Wheaton G, Zühlke L, Carapetis J (2012). World Heart Federation criteria for echocardiographic diagnosis of rheumatic heart disease–an evidence-based guideline. Nat Rev Cardiol.

[19] Census Office Of The State Council Of China National Bureau Of Statistics of China. Tabulation on the 2010 population census on the People's Republic of China. Beijing: China Statistics Press, 2012. http://www.Stats.Gov.Cn/tjsj/pcsj/rkpc/6rp/indexch.htm. 20 May 2012.

[20] Hu P, Liu X, Liang J, Zhu Q, Pu C, Tang M, Wang J (2017). A hospital-based survey of patients with severe valvular heart disease in China. Int J Cardiol.

[21] Iung B, Delgado V, Rosenhek R, Price S, Prendergast B, Wendler O, De Bonis M, Tribouilloy C, Evangelista A, Bogachev-Prokophiev A, Apor A, Ince H, Laroche C, Popescu BA, Piérard L, Haude M, Hindricks G, Ruschitzka F, Windecker S, Bax JJ, Maggioni A, Vahanian A, EORP VHD II Investigators (2019). Contemporary presentation and management of valvular heart disease: The EURObservational research programme valvular heart disease II survey. Circulation.

[22] Xinghe H, Jing L, Xueke B (2019). A hospital-based retrospective study of patients with valvular heart diseases in China (the China Peace retrospective study of valvular heart disease). JACC.

[23] Rwebembera J, Manyilirah W, Zhu ZW, Nabbaale J, Namuyonga J, Ssinabulya I, Lubega S, Lwabi P, Omagino J, Okello E (2018). Prevalence and characteristics of primary left-sided valve disease in a cohort of 15,000 patients undergoing echocardiography studies in a tertiary hospital in Uganda. BMC Cardiovasc Disord.

[24] Triki F, Jdidi J, Abid D, Tabbabi N, Charfeddine S, Ben Kahla S, Hentati M, Abid L, Kammoun S (2017). Characteristics, aetiological spectrum and management of valvular heart disease in a Tunisian cardiovascular centre. Arch Cardiovasc Dis.

[25] Yu Q, Wang B, Wang Y, Dai CL (2019). Level and trend of cardiovascular disease mortality in China from 2002 to 2016. Zhonghua Xin Xue Guan Bing Za Zhi.

[26] Chen HZ, Fan WH, Jin XJ, Wang Q, Zhou J, Shi ZY (2003). Changing trends of etiologic characteristics of cardiovascular diseases among inpatients in Shanghai: a retrospective observational study from 1948 to 1999. Zhonghua Nei Ke Za Zhi.

[27] Iung B, Baron G, Tornos P, Gohlke-Bärwolf C, Butchart EG, Vahanian A (2007). Valvular heart disease in the community: a European experience. Curr Probl Cardiol.

[28] Andell P, Li X, Martinsson A, Andersson C, Stagmo M, Zöller B, Sundquist K, Smith JG (2017). Epidemiology of valvular heart disease in a Swedish nationwide hospital-based register study. Heart.

[29] Yunqing Y, Haiyan X, Zhe L, Qingrong L, Xiling Q, Jie H, Yongjian W (2019). Distribution and etiology of valvular heart disease in the elderly in different regions of China. Chin J Geriatr Heart Brain Vessel Dis.

[30] Bekeredjian R, Grayburn PA (2005). Valvular heart disease. Circulation.

[31] Singh JP, Evans JC, Levy D (1999). Prevalence and clinical determinants of mitral, tricuspid, and aortic regurgitation (the Framingham Heart Study). Am J Cardiol.

[32] Baumgartner RN, Heymsfield SB, Roche AF (1995). Human body composition and the epidemiology of chronic disease. Obes Res.

[33] De Schutter A, Lavie CJ, Milani RV (2014). The impact of obesity on risk factors and prevalence and prognosis of coronary heart disease-the obesity paradox. Prog Cardiovasc Dis.

[34] Benjamin EJ, Muntner P, Alonso A, Bittencourt MS, Callaway CW, Carson AP, Chamberlain AM, Chang AR, Cheng S, Das SR, Delling FN, Djousse L, Elkind MSV, Ferguson JF, Fornage M, Jordan LC, Khan SS, Kissela BM, Knutson KL, Kwan TW, Lackland DT, Lewis TT, Lichtman JH, Longenecker CT, Loop MS, Lutsey PL, Martin SS, Matsushita K, Moran AE, Mussolino ME, O'Flaherty M, Pandey A, Perak AM, Rosamond WD, Roth GA, Sampson UKA, Satou GM, Schroeder EB, Shah SH, Spartano NL, Stokes A, Tirschwell DL, Tsao CW, Turakhia MP, VanWagner LB, Wilkins JT, Wong SS, Virani SS, American Heart Association Council on Epidemiology and Prevention Statistics Committee and Stroke Statistics Subcommittee (2019). Heart disease and stroke statistics-2019 update: a report from the American Heart Association. Circulation.

[35] Arden C, Chambers JB, Sandoe J, Ray S, Prendergast B, Taggart D, Westaby S, Grothier L, Wilson J, Campbell B, Gohlke-Bärwolf C, Mestres CA, Rosenhek R, Pibarot P, Otto CM (2014). Can we improve the detection of heart valve disease?. Heart.

[36] Celermajer DS, Chow CK, Marijon E, Anstey NM, Woo KS (2012). Cardiovascular Disease in the Developing World: prevalences, patterns, and the potential of early disease detection. JACC.

