# Supplementary material for: Current status and etiology of valvular heart disease in China: a population-based survey
Source: BMC Cardiovasc Disord. 2021 Jul 13;21:339. doi: 10.1186/s12872-021-02154-8 (PMC8276378; doi:10.1186/s12872-021-02154-8)
Supplement: Supplementary file 1 — Additional file 1 More results of the analysis. [file 12872_2021_2154_MOESM1_ESM.docx]

| S1Table1 Prevalence of valvular dysfunctions in right heart according to characteristics | | | | | | |
| --- | --- | --- | --- | --- | --- | --- |
| Characters |  | Number | TS | TR | PS | PR |
| Total |  | 31,499 | 71.6(19.3-265.1) | 841.1(436.9-1613.4) | 1.5(0.1-15.0) | 118.5(47.9-293.3) |
| Gender | Male | 14,470 | 91.0(20.1-411.2) | 629.2(378.8-1043.4) | 0 | 98.6(40.7-238.6) |
|  | Female | 17,029 | 51.6(17.4-152.8) | 1059.4(489.9-2275.6) | 3.0(0.3-28.2) | 139.1(51.5-375.0) |
| *P* |  |  | 0.277 | 0.017 | - | 0.254 |
| Age (years) | 35-44 | 6,849 | 78.8(24.7-251.2) | 359.2(138.2-929.8) | 0 | 18.7(3.9-89.5) |
|  | 45-54 | 7,406 | 41.3(7.4-229.4) | 459.7(231.4-911.0) | 0 | 99.9(36.9-270.2) |
|  | 55-64 | 6,765 | 120.6(28.5-508.2) | 1020.4(447.8-2307.9) | 0 | 96.7(34.9-267.8) |
|  | 65-74 | 6,406 | 0 | 1577.2(792.1-3115.9) | 0 | 166.3(52.1-529.1) |
|  | 75- | 4,073 | 132.9(35.1-501.7) | 3967.0(2228.0-6966.8) | 27.8(4.5-172.3) | 881.1(308.0-2493.9) |
| *P* |  |  | - | <0.001 | - | <0.001 |
| BMI (Kg/m^2^) | <18.5 | 1,319 | 18.2(1.0-325.6) | 1043.5(476.7-2268.4) | 18.2(1.0-325.6) | 289.2(76.0-1093.3) |
|  | 18.5-23.9 | 13,036 | 56.1(17.5-179.3) | 1137.0(564.5-2276.9) | 1.5(0.1-32.4) | 180.3(65.5-495.5) |
|  | 24-27.9 | 12,042 | 122.8(27.9-538.0) | 630.7(334.8-1184.9) | 0.6(0.0-33.1) | 37.2(14.5-95.6) |
|  | 28< | 5,102 | 0 | 543.2(211.8-1386.1) | 0 | 118.8(34.6-407.4) |
| *P* |  |  | - | 0.005 | - | 0.012 |
| Region | East | 14,009 | 1.6(0.1-30.4) | 892.3(525.2-1512.2) | 0 | 85.2(38.2-189.6) |
|  | Central | 11,895 | 0.8(0.0-33.8) | 186.5(89.2-389.3) | 0 | 168.2(25.1-1116.4) |
|  | West | 5,595 | 330.7(125.5-868.6) | 1793.2(593.0-5292.8) | 6.9(0.4-113.9) | 107.2(49.2-233.8) |
| *P* |  |  | <0.001 | <0.001 | - | 0.683 |
| Residence | Rural | 15,009 | 146.9(31.5-683.1) | 1285.0(513.5-3178.8) | 3.9(0.5-33.0) | 111.5(67.6-183.9) |
|  | Urban | 16,490 | 26.4(7.7-89.8) | 575.0(291.4-1131.4) | 0 | 122.7(31.0-483.9) |
| *P* |  |  | 0..013 | 0.081 | - | 0.898 |
| Hypertension | No | 18,163 | 51.3(13.9-189.5) | 704.4(359.4-1376.0) | 1.3(0.1-23.7) | 72.1(30.0-172.9) |
|  | Yes | 13,336 | 109.0(28.2-420.0) | 1094.1(579.6-2056.0) | 1.8(0.1-32.2) | 204.5(73.1-571.0) |
| *P* |  |  | 0.006 | <0.001 | <0.001 | 0.007 |
| Dyslipidemia | No | 21,830 | 56.0(18.0-174.2) | 958.2(546.7-1674.4) | 2.2(0.2-21.7) | 130.7(54.1-315.7) |
|  | Yes | 9,669 | 104.0(21.9-491.5) | 596.7(211.0-1675.5) | 0 | 93.1(33.0-262.8) |
| *P* |  |  | 0.131 | 0.145 | - | 0.136 |
| Diabetes | No | 28,214 | 78.2(21.1-289.7) | 844.0(440.7-1610.4) | 1.6(0.2-16.7) | 116.8(48.0-284.1) |
|  | Yes | 9,669 | 0 | 810.7(358.0-1825.1) | 0 | 137.3(37.5-501.7) |
| *P* |  |  | - | 0.853 | - | 0.717 |
| Stroke | No | 30,656 | 72.7(19.7-268.6) | 817.9(420.0-1586.7) | 1.5(0.1-15.4) | 116.0(45.7-294.1) |
|  | Yes | 843 | 0 | 2287.5(1103.8-4680.5) | 0 | 276.2(56.7-1334.4) |
| *P* |  |  | - | 0.004 | - | 0.347 |
| CKD | No | 29,453 | 56.8(16.6-194.1) | 783.9(398.3-1537.1) | 1.5(0.1-16.0) | 113.7(46.1-280.2) |
|  | Yes | 2,046 | 380.7(77.7-1844.7) | 2038.7(1108.3-3720.9) | 0 | 219.0(72.8-656.8) |
| *P* |  |  | <0.001 | <0.001 | - | 0.039 |
| Prevalence were reported as number×10－^3^ percentage (95% CI).  All values were weighted to represent the total population of Chinese aged 35 years or older based on Chinese census 2010.  Abbreviation: TS= tricuspid stenosis; TR= tricuspid regurgitation; PS= pulmonary stenosis; PR= pulmonary regurgitation; BMI= body mass index; CKD= chronic kidney disease.  *P*-value for x^2^ or Fisher’s exact test to assess the difference of prevalence among subgroups according to the characteristics. | | | | | | |

| S1Table2 The component proportions of etiologies of valvular heart disease according to age | | | | | | |
| --- | --- | --- | --- | --- | --- | --- |
|  | Congenital anomaly | Degenerative | Others | Rheumatic | Secondary | Valve replacement |
| 35-44 | 1.6(0.3-1.0) | 0.0(0.0-0.0) | 8.0(3.9-15.5) | 77.8(66.9-85.8) | 12.3(5.0-27.1) | 0.3(0.0-4.7) |
| 45-54 | 0.1(0.0-3.6) | 0.0(0.0-0.0) | 9.9(4.7-17.4) | 70.0(56.3-83.8) | 16.3(8.9-28.0) | 3.7(1.2-10.3) |
| 55-64 | 1.5(0.5-4.8) | 18.2(9.5-32.1) | 11.6(7.2-16.7) | 55.1(45.1-65.1) | 12.3(6.7-21.5) | 1.3(0.4-4.0) |
| 65-74 | 0.0(0.0-0.0) | 35.8(25.6-46.0) | 9.9(7.1-13.5) | 43.4(31.9-55.0) | 10.8(7.9-14.8) | 0.1(0.0-1.2) |
| 75- | 0.0(0.0-0.0) | 42.5(35.9-48.8) | 9.7(6.2-14.1) | 38.0(32.0-43.9) | 9.8(5.6-16.6) | 0.1(0.0-0.9) |
| Total | 0.7(0.2-2.0) | 21.3(15.3-28.7) | 9.9(7.8-11.9) | 55.1(51.0-59.2) | 12.1(7.6-18.6) | 0.9(0.4-2.1) |
| Data were represented as percentage (95% CI).  All values were weighted to represent the total population of Chinese aged 35 years or older based on Chinese census 2010. | | | | | | |


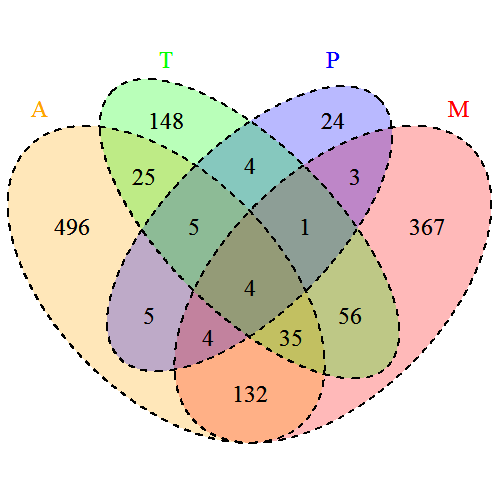


S1Figure1 Overlap of patients with single or multiple valvular. P, pulmonary valvular dysfunction; T, tricuspid valvular dysfunction; M, mitral valvular dysfunction; A, aortic valvular dysfunction.
