# Supplementary material for: Current status and etiology of valvular heart disease in China: a population-based survey
Source: BMC Cardiovasc Disord. 2021 Jul 13;21:339. doi: 10.1186/s12872-021-02154-8 (PMC8276378; doi:10.1186/s12872-021-02154-8)
Supplement: Supplementary file 2 — Additional file 2 The full list of work staff for the survey. [file 12872_2021_2154_MOESM2_ESM.docx]

**S2 file1: List of investigators of the China Hypertension Survey Study**

This study was accomplished through the fine work of the staff at the national level. For a partial listing of colleagues see the follows (provinces sorted as alphabetical order):

**Anhui**: Liqun Hu, Hongqi Li, Qi Zhang, Guang Yan, Anhui Provincial Hospital, Hefei, Anhui, China; Fangfang Zhu, Anhui Institute of Cardiovascular Disease, Hefei, Anhui, China.

**Beijing**: Xianghua Fang, Chunxiu Wang, Shaochen Guan, Xiaoguang Wu, Hongjun Liu, Chengbei Hou, Xuanwu Hospital, Capital Medical University, Beijing, China.

**Chongqing**: Han Lei, Wei Huang, Nan Zhang, First Affiliated Hospital of Chongqing Medical University, Chongqing, China; Ge Li, Lihong Mu, Xiaojun Tang, Chongqing Medical University, Chongqing, China.

**Fujian**: Ying Han, Huajun Wang, Dongjie Lin Liangdi Xie, First Affiliated Hospital of Fujian Medical University, Fuzhou, Fujian, China; Daixi Lin, Fujian medical university, Fuzhou, Fujian, China.

**Gansu**: Jing Yu, Xiaowei Zhang, Wei Liang, Heng Yu, Qiongying Wang, Lanzhou University Second Hospital, Lanzhou, Gansu, China; Lan Yang, Maternal and Child Care Service Centre, Lanzhou, Gansu, China.

**Guangdong**: Yingqing Feng, Yuqing Huang, Guangdong General Hospital, Guangzhou, Guangdong, China; Peixi Wang, Jiaji Wang, Guangzhou Medical University, Guangzhou, Guangdong, China; Harry HX Wang, Sun Yat-Sen University, Guangzhou, Guangdong, China; Songtao Tang, Community Health Services Center of Liaobu, Dongguan, Guangdong, China.

**Guangxi**: Tangwei Liu, Rongjie Huang, Zhiyuan Jiang, Haichan Qin, First Affiliated Hospital of Guangxi Medical University, Nanning, Guangxi, China.

**Guizhou**: Guoqin Liu, Zhijun Liu, Wenbo Rao, Zhen Chen, Yalin Chu, Fang Wu, Zunyi Medical University, Zunyi, Gouzhou, China.

**Hainan**: Haitao Li, Jianlin Ma, Tao Chen, Hainan General Hospital, Haikou, Hainan, China; Ming Wu, Health and Family Planning Commission of Hainan, Haikou, Hainan, China.

**Hebei**: Jixin Sun, Yajing Cao, Yuhuan Liu, Center for Disease Prevention and Control of Hebei, Shijiazhuang, Hebei, China; Zhikun Zhang, Center for Disease Prevention and Control of Tangshan, Tangshan, Hebei, China; Yanmei Liu, Center for Disease Prevention and Control of Langfang, Langfang, Hebei, China; Dejin Dong, Center for Disease Prevention and Control of Xingtai, Xingtai, Hebei, China; Guangrong Li, Center for Disease Prevention and Control of Dingzhou, Dingzhou, Hebei, China.

**Heilongjiang**: Hong Guo, Lihang Dong, Haiyu Zhang, Fengyu Sun, Xingbo Gu, Ye Tian, First Affiliated Hospital of Harbin Medical University, Haerbin, Heilongjiang, China.

**Henan**: Kaijuan Wang, Chunhua Song, Peng Wang, Hua Ye, Zhengzhou University, Zhengzhou, Henan, China; Wei Nie, Shuying Liang, Henan Academy of Medical Sciences, Zhengzhou, Henan, China.

**Hubei**: Congxin Huang, Fang Chen, Yan Zhang, Heng Zhou, Jing Xie, Jianfang Liu, Department of Cardiology, Renmin Hospital of Wuhan University, Wuhan, Hubei, China.

**Hunan**: Hong Yuan, Chengxian Guo, Third Xiangya Hospital, Central South University, Changsha, Hunan, China; Yuelong Huang, Biyun Chen, Center for Disease Control and Prevention of Hunan, Changsha, Hunan, China.

**Inner Mongolia**: Xingsheng Zhao, Wenshuai He, Xia Wen, Yanan Lu, Inner Mongolia people's hospital, Hohhot, Inner Mongolia, China.

**Jiangsu**: Xiangqing Kong, Ming Gui, Wenhua Xu, Yan Lu, Jun Huang, First Affiliated Hospital of Nanjing Medical University, Nanjing, Jiangsu, China; Min Pan, Affiliated Hospital of Nantong University, Nanjing, Jiangsu, China; Jinyi Zhou, Ming Wu, Center for Disease Control and Prevention of Jiangsu, Nanjing, Jiangsu, China.

**Jiangxi**: Xiaoshu Cheng, Huihui Bao, Xiao Huang, Kui Hong, Juxiang Li, Ping Li, Second Affiliated Hospital of Nanchang University, Nanchang, Jiangxi, China.

**Jilin**: Bin Liu, Junduo Wu, Longbo Li, Yunpeng Yu, Yihang Liu, Chao Qi, Second Hospital of Jilin University, Changchun, Jilin, China.

**Liaoning**: Jun Na, Li Liu, Yanxia Li, Guowei Pan, Center for Disease Prevention and Control of Liaoning, Shenyang, Liaoning, China; Degang Dong, Peng Qu, Health and Family Planning Commission of Liaoning, Shenyang, Liaoning, China.

**Ningxia**: Jinbao Ma, Health and Family Planning Commission of Ning Xia Hui Autonomous Region, Yinchuan, Ningxia, China; Juan Hui, Center for Disease Control and Prevention of Ning Xia Hui Autonomous Region, Yinchuan, Ningxia, China; Fu Zhao, Health Supervision Institute of Xixia District in Yinchuan, Ning Xia Hui Autonomous Region, Yinchuan, Ningxia, China.

**Qing Hai**: Jianning Yue, Minru Zhou, Zhihua Xu, Xiaoping Li, Qiongyue Sha, Fuchang Ma, Qing Hai Center for Disease Control and Prevention, Xining, Qinghai, China; Qiuhong Chen, Huiping Bian, Qinghai Cardio-Cerebrovascular Disease Special Hospital, Xining, Qinghai, China.

**Shaanxi**: Jianjun Mu, Tongshuai Guo, Keyu Ren, Chao Chu, First Affiliated Hospital of Xi’an Jiaotong University, Xian, Shaanxi, China.

**Shandong**: Zhendong Liu, Hua Zhang, Yutao Diao, Shangwen Sun, Yingxin Zhao, Institute of Basic Medicine, Shandong Academy of Medical Sciences, Jinan, Shandong, China.

**Shanghai**: Junbo Ge, Jingmin Zhou, Xuejuan Jin, Jun Zhou, Zhongshan Hospital, Fudan University, Shanghai, China.

**Shanxi**: Bao Li, Lijun Zhu, Yuean Zhang, Gang Wang, Shanxi Cardiovascular Hospital, Taiyuan, Shanxi, China; Zhihan Hao, Wuxiang County People's Hospital, Wuxiang, Shanxi, China.

**Sichuan**: Li Cai, Zhou Liu, Zhengping Yong, Jianhong Tao, Yijia Tang, Sichuan Provincial People's Hospital, Chengdu, Sichuan, China; Shaoping Wan, Sichuan Cancer Hospital, Chengdu, Sichuan, China.

**Tianjin**: Zhenshan Jiao, Yuqiang Fan, Tianjin Academy of Traditional Chinese Medicine, Tianjin, China; Hui Gao, Wei Wang, Tianjin Municipal Commission of Health and Family Planning, Tianjin, China; Qingkui Li, Xiaomei Zhou, Tianjin Medical University, Tianjin, China.

**Tibet**: Yundai Chen, Bin Feng, Qinglei Zhu, Sansan Zhou, Chinese People’s Liberation Army General Hospital, Lasha, Tibet, China.

**Xinjiang**: Nanfang Li, Lin Zhou, Delian Zhang, Jing Hong, People's Hospital of Xinjiang Uygur Autonomous Region, Urumuqi, Xinjiang, China.

**Yunnan**: Tao Guo, Min Zhang, First Affiliated Hospital of Kunming Medical University, Kunming, Yunnan, China; Yize Xiao, Center for Disease Prevention and Control of Yunnan, Kunming, Yunnan, China; Xuefeng Guang, Affiliated Yan'an Hospital of Kunming Medical University, Kunming, Yunnan, China.

**Zhejiang**: Xinhua Tang, Jing Yan, Xiaoling Xu, Li Yang, Aimin Jiang, Wei Yu, Zhejiang Hospital, Hangzhou, Zhejiang, China.

**S2 file2: The Steering Committee of China Hypertension Survey (CHS):**

**Leading Group:**

- Leader: Wei He
- Deputy:

Lingzhi Kong, Lisheng Liu, Liming Li, Shengshou Hu, Runlin Gao

- Members:

Guofu Ding, Hao Yu, Weihang Ma, Lan Wu, Xiangqing Kong, Hui Wang, Zhengrong Wang, Zaitao Wang, Songling Wang, Xiaojuan Wang, Feng Lan, Lin Gan, Ahemaitijiang Yishake, Yueqi Wu, Wei Liu, Lin Liu, Kui Liu, Zhongzheng Liu, Yuchi Liu, Guohua Liu, Guisheng Liu, Shuqun Lu, Ningsheng Sun, Xiaojun Sun, Pengli Zhu, Shihu Jiang, Ronghui Jiang, Jichun Xing, Jiansheng Yu, Ming Wu, Mei Song, Ling Zhang, Jin Zhang, Kan Zhang, Guangqi Zhang, Kaijun Zhang, Jianguo Zhang, Xingbiao Shi, Jingfeng Li, Zhigang Li, Yuejian Li, Jianguang Li, Chunhong Li, Luping Li, Zhonglai Yang, Chaokuan Yang, Lin Su, Lin Lu, Jie Chen, Li Chen, Xiaochun Chen, Yuansheng Chen, Yongzhong Chen, Xiuhua Chen, Jianzhong Chen, Ce Zhou, Ping Yue, Jun Luo, Lining Miao, Zhenxian Fan, Jin Zheng, Kui Hong, Guangguo Zhao, Lei Zhong, Qingfeng Xu, Shuping Yuan, Yufeng Guo, Hui Gao, Qifeng Cao, Xiaoyuan Cao, Lisha Zhang, Wei Huang(Henan province), Lixun Huang, Xingli Huang, Fengping Huang, Wei Peng, Zhiyu Zeng, Xiaoshu Cheng, Degang Dong, Yunlong Xie, Liangdi Xie, Yuan Xie, Wuxu Chao, Yongqian Zhai.

**China CHS Study Advisory Committee:**

- Leader: Lisheng Liu
- National members:

Shuping Ma, Haiyan Wang, Dingliang Zhu, Baohua Ji, Kegui Wu, Yinsheng Wu, Xigui Wu, Tingjie Zhang, Yude Chen, Deyu Zheng, Hongyi Jin, Zhaoguang Hong, Lianyou Zhao, Shiying Fu, Changyu Pan.

- International members:

G. Fodor, Minto Prevention and Rehabilitation Centre, University of Ottawa, Heart Institute, Canada;

S. Mendis, Coordinator, Cardiovascular Diseases. World Health Organization

A. Chockalingam, Office of Global Health, NHLBI, NIH, USA;

J. Staessen, Studies Coordinating Centre, Laboratory of Hypertension, Leuven University, Belgium;

Kiang Liu, Department of Preventive Medicine, Feinberg School of Medicine, Northwestern University, USA;

Yuanli Liu, Department of Global Health and Population, Harvard School of Public Health, USA;

Jing Ma, Department of Medicine, Brigham and Women's Hospital and Harvard Medical School, USA;

Lu Tian, Department of Health Research and Policy, Stanford University School of Medicine, USA;

Zugui Zhang, Director of Biostatistics, Christiana Care Health System, USA

William S Weintraub, Director of Clinical Outcomes Research, MedStar Washington Hospital Center, Washington DC., USA.

**China CHS Study Expert Committee:**

- Leader: Runlin Gao
- Deputy:

Jiguang Wang, Zengwu Wang, Zhaosu Wu, Yun Zhang, Shu Zhang, Xiaoying Li, Guangwei Li, Xiaomei Li, Shuigao Jin, Dayi Hu, Jun Huang, Junbo Ge.

- Members:

Aitian Yin, Ruixing Yin, Xianghua Fang, Jinbao Ma, Wen Wang, Zhong Wang, Wei Wang, Xiaowan Wang, Kaijuan Wang, Jianan Wang, Hao Wang (Henan province), Hao Wang (Fuwai Hospital), Jiaji Wang, Yanping Wang, Jingfeng Wang, Changjun Tian, Tian Ye (Heilongjiang province), Feng Bai, Kaichao Long, Jianghua Ren, Xiaolan Ren, Xuefeng Guang, Bin Liu, Guozhang Liu, Guohua Liu, Guoqin Liu, Jianhua Liu, Zhiquan Liu, Tangwei Liu, Jiafa Liu, Dianwu Liu, Qi Hua, Xiuzhang Lu, Guang Sun, Ningling Sun, Yingxian Sun, Ping Qu, Liguang Zhu, Lijun Zhu, Jianhua Zhu, Manlu Zhu, Hong Jiang, Shihu Jiang, Ronghui Jiang, Baopeng Tang, Jianjun Mou, Suhua Yan, Qinyun Ruan, Xiaoyong Qi, Guang Yan, Jing Yan, Ji Yan, Yao He, Jing Yu, Zhenqiu Yu, Shulin Wu, Kegui Wu, Lirong Wu, Jian Zhang, Xiaowei Zhang, Shijie Zhang, Lizhen Zhang, Kaijun Zhang, Jianxin Zhang, Yanjie Zhang, Bao Li, Ling Li, Lang Li, Bin Li, Guangping Li, Weiming Li, Zhanquan Li, Shoujun Li, Chengling Li, Jiangrong Li, Yongjun Li, Nanfang Li, Shumei Li, Tianlun Yang, Tianhe Yang, Donghui Yang, Yuejin Yang, Chaokuan Yang, Daowen Wang, Yize Xiao, Weiwei Chen, Jiyan Chen, Yundai Chen, Jingmin Zhou, Danhui Yi, Xine Yi, Xue Lin, Shuguang Lin, Jian Wu, Xing Yuan, Fanghong Lu, Zhenshan Jiao, Zhenxian Fan, Xinhui Jin, Yun Yao, Chonghua Yao, Jun Hong, Chuanlai Hu, Dong Zhao, Wenhua Zhao, Yujuan Zhao, Xingsheng Zhao, Yarong Hao, Guoqiang Zhong, Xinhua Tang, Chaowu Xu, Xinjuan Xu, Luobu Gesang, Ming Gui, Feiya Suo, Hong Yuan, Zuyi Yuan, Tao Guo, Wei Guo, Guizhou Tao, Dongfeng Gu, Yueqin Gao, Chuanyu Gao, Qianhui Shang, Congxin Huang, Yonglin Huang, Dejia Huang, Xiaoshu Cheng, Liangdi Xie, Jiuhai Han, Han Lei, Yuhua Liao, Li Cai, Lin Cai, Yunchang Cai.

**China CHS Study Steering Committee:**

- Leader: Runlin Gao
- Deputy: Zengwu Wang
- Members:

Linqi Diao, Yutao Diao, Lianzheng Yu, Shaoping Wan, Jianlin Ma, Xiangqing Kong, Zhanchun Yin, Xianghua Fang, Sheng Wang, Peng Wang, Zhong Wang, Wei Wang, Xin Wang, Xiaowan Wang, Huajun Wang, Lianzhong Wang, Jiaji Wang, Haoxiang Wang, Jingjing Feng, Bin Feng, Yingqing Feng, Yiping Feng, Li Zuo, Meng Tian, Ye Tian (Fuwai Hospital), Huiping Bian, Xiaolan Ren, Bing Liu, Xiaoqing Liu, Huzi Liu, Jiafa Liu, Guoqin Liu, Zhihua Liu, Zhendong Liu, Dianwu Liu, Ningsheng Sun, Jixin Sun,SHangwen Sun, Lijun Zhu, Baoyu Zhu, Junqing Zhu, Jianhua Zhu, Zhenhui Zhu, Pengli Zhu, Ronghui Jiang, Jianjun Mou, Jun Na, Lin He, Xinye He, Hua Yu, Jing Yu, Yun Wu, Xiaoying Wu, Xiaowei Zhang, Yuean Zhang, Yonghui Zhang, Yuqing Zhang, Yuhui Zhang, Jianxin Zhang, Linfeng Zhang, Shujuan Zhang, Yuanming Zhang, Qingkui Li, Xian Li, Bao Li, Ge Li, Man Li, Guoju Li, Nanfang Li, Donghui Yang, Jingyuan Yang, Ruiying Yang, Jingyuan Yang, Lan Shao, Yang Chen, Zuo Chen, Zhen Chen, Hui Chen, Guanliang Chen, Hong Chen, Shulan Chen, Yundai Chen, Jingzhuang Mai, Songtao Tang, Ciren zhuoma, Xiaomei Zhou, Jun Zhou, Ling Zhou, Jinyi Zhou, Minru Zhou, Wenbo Rao, Xine Yi, Jianning Yue, Xue Lin, Suxin Luo, Xuejuan Jin, Xingfang Jin, Jun Hong, Kui Hong, Liqun Hu, Wenshuai He, Liping He, Xingsheng Zhao, Yingxin Zhao, Guang Hao, Yarong Hao, Zhengchun Niu, Xinhua Tang, Yuqiang Fan, Xiaoling Xu, Wenhua Xu, Guoyan Xu, Qingbin Xu, Ming Gui, Wei Nie, Hong Yuan, Qinhui Jia, Hong Guo, Tao Guo, Ying Guo, Rui Guo, yongjun Guo, Min Guo (Fuwai Hospital), Yueying Cui, Juan Hui, Shuying Liang, Kai Huang, Wei Huang (Chongqing), Feng Huang, Congxin Huang, Rongjie Huang, Yanhong Huang, Zhiyu Zeng, Xiaojun Wen, Lihang Dong, Weihong jiang, Liangdi Xie, Ying Han, Minghua Han, Lin Cai, Guowei Pan.

- - **Quality control group**

Lianzheng Yu, Xin Ma, Wen Ma, Peng Wang, Xin Wang, Lianzhong Wang, Zengwu Wang, Meng Tian, Ye Tian (Fuwai Hospital), Jingjing Feng, Baoyu Zhu, Junqing Zhu, Manlu Zhu, Xiaolan Ren, Xi Liu, Bin Liu, Xiaoqing Liu, Mingbo Liu, Jingzhuang Mai, Heping Hua, Wei Li, Ge Li, Ping Li, Man Li, Guohua Li, Guoju Li, Puren Li, Xiaohui Yang, Lirong Wu, Xinye He, Hua Yu, Yuean Zhang, Linfeng Zhang, Yang Chen, Zuo Chen, Weiwei Chen, Guanliang Chen, Yude Chen, Lan Shao, Ciren Zhuoma, Shuigao Jin, Xuejuan Jin, Guang Hao, Chonghua Yao, Liping He, Ming Gui, Rui Guo, Min Guo (Fuwai Hospital), Min Guo (Xinjiang), Haiqin Tang, Xinhua Tang, Bu Sang, Yanhong Huang, Weihua Cao, Yueying Cui, Yimu Liang, Lihang Dong, Weihong Jiang, Minghua Han, Yongqian Zhai.

- - **Data Management Group**

Qiangxiao Ding, Wenying Wang, Lijun Wang, Lianzhong Wang, Chunxiu Wang, Jingjing Feng, Lijian Sheng, Xi Liu, Jianhua Liu, Huang Sun, Jun Na, Hua Yu, Xiaoguang Wu, Xiaowei Zhang, Yuean Zhang, Wei Li, Guoju Li, Haitao Li, Zheng Yang, Jingyuan Yang, Zuo Chen, Lijuan Chen, Yude Chen, Jingzhuang Mai, Jun Zhou, Shuigao Jin, Guang Hao, Zhengchun Niu, Xiaoling Xu, Qingbin Xu, Ming Gui, Shiyong Qin, Rui Guo, Min Guo (Xinjiang), Yueying Cui, Wei Huang (Chongqing), Zhijun Huang, Yanhong Huang, Feng Peng, Zhiyuan Jiang.

- - **Project Coordinating Office**

Lan Shao, Min Guo (Fuwai Hospital), Ye Tian (Fuwai Hospital), Yang Chen, Jie Ma, Xinye He, Meng Tian, Xiuyun Jia.
